# Effects of Polystyrene Nanoplastics on Oxidative Stress, Blood Biochemistry, and Digestive Enzyme Activity in Goldfish (*Carassius auratus*)

**DOI:** 10.3390/toxics13050336

**Published:** 2025-04-24

**Authors:** Sasan Azarm-Karnagh, Masoud Sattari, Mahdi Banaee, Behzad Shirkavand Hadavand, Francesca Falco

**Affiliations:** 1 Fisheries Department, Faculty of Natural Resources, University of Guilan, Sowmeh Sara P.O. Box 1144, Iran; sasanazarm721@gmail.com; 2 Aquaculture Department, Faculty of Natural Resources, Behbahan Khatam Alanbia University of Technology, Behbahan P.O. Box 63616-64973, Iran; mahdibanaee2@gmail.com; 3 Department of Resin and Additives, Institute for Color Science and Technology, Tehran P.O. Box 16765-654, Iran; b.shirkavand@gmail.com; 4 Institute for Biological Resources and Marine Biotechnologies (IRBIM), 91026 Mazara del Vallo, Italy

**Keywords:** polystyrene nanoplastics, reactive oxygen species (ROS), integrated biomarker response (IBR), aquatic toxicity, environmental pollution

## Abstract

Plastic pollution in aquatic ecosystems has become a global concern, and nanoplastics, due to their small size and high penetration potential, pose significant risks to aquatic organisms. This study aimed to evaluate the effects of polystyrene nanoplastics (PS-NPs) on oxidative stress biomarkers, blood biochemical parameters, and digestive enzyme activities in Goldfish (*Carassius auratus*). The experiment was conducted over 21 days, exposing fish to four different dietary concentrations of PS-NPs (0, 1, 10, and 100 mg/Kg). The results showed that chronic exposure to 50 nm PS-NPs significantly increased the activity of antioxidant enzymes (CAT, SOD, and GPx) and MDA activity in the gill, kidney, and liver tissues, indicating the induction of oxidative stress. Furthermore, notable alterations were observed in blood biochemical enzymes (alkaline phosphatase [ALP], alanine aminotransferase [ALT], aspartate aminotransferase [AST], and lactate dehydrogenase [LDH]), suggesting cellular damage and physiological disturbances. Additionally, PS-NP exposure affected the activity of digestive enzymes (amylase, lipase, and protease), which may impact nutrient digestion and absorption. These findings highlight that PS-NPs can adversely affect aquatic animal health and may influence the nutritional quality of fish. Therefore, further research is necessary to assess the long-term ecological and toxicological impacts of nanoplastics in freshwater ecosystems.

## 1. Introduction

Plastic pollution in aquatic ecosystems is a worldwide issue and has increasingly drawn attention from the scientific community recently [1,2,3]. Discarded plastics are broken down by biotic factors, such as digestion [4], and abiotic factors, such as UV radiation [5]. These processes degrade plastics into microplastics (MPs) (particles smaller than 5 mm) [6] and nanoplastics (NPs) (particles smaller than 100 nm) [7]. Although current environmental concentrations of nanoplastics are not yet well documented due to analytical limitations [8], their widespread presence in aquatic environments has been strongly emphasized [9]. As particle size decreases, plastic becomes more bioavailable and potentially more hazardous to aquatic organisms [10]. Due to their nanoscale properties, including enhanced mobility and high surface area, NPs may be easily ingested by aquatic organisms [11]. They can also penetrate tissues [12] and accumulate in several organs and tissues [13]. Aquatic organisms can take NPs from the environment through direct ingestion [14,15] as well as through trophic transfer along the food chain, thereby potentially affecting top consumers, including humans [16,17,18,19].

Under laboratory conditions, NPs have been observed to enter fish organs mainly through the gills and digestive tract [20,21], after which they may be distributed to other organs via the circulatory system and subsequently accumulate [22]. A growing body of evidence indicates that NPs can induce oxidative stress [23,24], immune dysfunction [15,25], teratogenic effects [26,27], altered locomotor behavior [16,17,28], and impaired feeding behavior [29]. In addition, altered lipid metabolism [17,18,30] and histological alterations [15,16] have been reported in fish exposed to the particles. Studies have confirmed that NPs have the potential to cause many adverse effects in fish, including inhibition of growth performance [31,32], early embryonic development [33], and oxidative stress [34,35], as well as affect the composition and relative abundance of the gut flora [32,36]. Tallec et al. (2018) and Yin et al. (2021) reported that NPs cause more significant neurotoxicity than MPs in shellfish [37,38]. Studies have shown that these nanoparticles can cross the blood–brain barrier in both fish [22] and mammals [39]. Therefore, studying polystyrene nanoplastics (PS-NPs) in fish brains can clarify their potential risks to human health and well-being. Digestive enzymes are also critical for the hydrolysis of proteins, carbohydrates, and lipids, which are commonly used as markers of bio-toxicity and digestive capacity [40,41,42,43].

Most research on PS-NPs has focused on zebrafish (*Danio rerio*), while relatively few studies have investigated other fish species [44]. However, given relevant factors, such as the cost of rearing and maintenance, commercial availability, genetic distance to the cultured species, and housing facilities, Goldfish (*Carassius auratus*) appear to be an ideal model organism to investigate the effects of NPs [45,46]. Although ontogenetic, intestinal, metabolic, or behavioral abnormalities have been previously reported in some species [32,47,48], the effects of chronic exposure to NPs in fish are still poorly understood. Polystyrene (PS) is one of the most widely used plastic polymers globally due to its easy availability. It is often used to examine the toxic effects of NPs in aquatic animals [15]. It is also one of the plastic polymers reported in aquatic environments [49,50]. A growing number of scientific studies have focused on the effects of PS-NPs on the health and performance of aquatic organisms such as fish [31,44,51]. PS, one of the most common plastics, is used to make disposable containers, foam boxes, and insulation materials [52]. Due to its low recycling rate, PS is commonly detected in aquatic environments [30,53]. Despite growing interest in PS-NP toxicity, significant knowledge gaps remain concerning its chronic effects on fish physiology—particularly in species such as Goldfish, which are broadly distributed in freshwater ecosystems and offer valuable insights as model organisms for ecotoxicological research. The novelty of this research lies in its comprehensive assessment of PS-NP toxicity across multiple physiological systems—oxidative stress, blood biochemistry, and digestive function—in Goldfish exposed to environmentally relevant and higher concentrations under chronic exposure during a 21-day period. We hypothesized that chronic dietary exposure to PS-NPs would induce dose-dependent oxidative stress, disrupt blood biochemical parameters, and impair digestive enzyme activities in Goldfish, reflecting broader physiological damage. Specifically, this study aimed to (1) evaluate the extent of oxidative stress in critical organs (liver, kidney, and gills) by measuring antioxidant enzyme activity and lipid peroxidation, (2) assess blood biochemical markers as indicators of systemic and cellular damage, and (3) determine the impact of PS-NPs on digestive enzyme activity as a proxy for nutrient assimilation and overall nutritional health. These objectives were driven by the need to understand how PS-NPs, as a prevalent aquatic pollutant, affect fish health beyond acute exposure scenarios and to elucidate their potential implications for freshwater ecosystems and food safety. By focusing on Goldfish, this research bridges a gap in species-specific data and provides a foundation for assessing the ecological and toxicological risks of NPs in a broader context. Analytical validation of biomarkers in measuring the effects of PS-NPs on the studied species is one of the secondary objectives of this research.

This study provides a better understanding of the effects of NPs on fish physiological health. Its results may also increase our knowledge about the adverse effects of NPs on fish nutritional quality.

## 2. Materials and Methods

### 2.1. NPs and Characterization

This study used a commercial PS-NP suspension (size 50 nm; Color Research Institute, Tehran, Iran). The suspension was stored at 4 °C in the dark. The PS-NP suspension was treated with an ultrasonic cleaner, MSE PRO 3L CNC (Tucson, AZ, USA; frequency 40 kHz, power 100 W, for 10 min) before each use [8] to ensure homogeneity and disaggregate any nanoparticle clumps that may have formed during storage at 4 °C, while minimizing the removal of inherent stabilizers present in the commercial suspension. This step was not intended to remove chemical additives such as stabilizers or dispersants but rather to maintain a uniform particle distribution for consistent exposure conditions. To determine the hydrodynamic size of the nanoparticles, a dynamic light scattering (DLS) device MicroTrace-NANOFlex (Haan, NRW, Germany; Table S1) and zeta potential (MicroTrace-Zeta Check) were used in ultrapure water. The size and morphology of the plastic were confirmed using a scanning electron microscope PHILIPS-XL30 (Montgomeryville, PA, USA; Figure S2). The chemical composition of the nanoparticles was determined using μ-FT-IR, LabRAM Aramis IR2–Horiba Scientific (Kyoto, Japan) (Figure S2).

### 2.2. NP-Diet Preparation

Four experimental diets were supplemented with PS-NPs at 0, 1, 10, and 100 mg/kg, respectively. In this study, a formulated diet (45% crude protein and 7% crude fat; Tropical-Goldfish color pellet, Poland) was used to prepare the experimental feed. The remaining 48% of the diet likely comprised carbohydrates, ash, moisture, and fiber, consistent with standard commercial Goldfish feed formulations, although the exact proportions were not analyzed [54]. The PS-NPs were incorporated into a commercial diet (Tropical-Goldfish color pellet Co., Ltd., Kalisz, Poland) with a size of 1–2 mm [54]. For the control diet, the food pellets were mixed with ultrapure water, and in the NP treatments, the suspension was mixed with 1, 10, and 100 mg/kg of PS-NP nanoparticles. The suspension (or ultrapure water) was added to 300 g of the diet and mixed thoroughly with a commercial food mixer, GOSONIC-GSM-905, 2021 (Tehran, Iran). A solution of 3 g of bovine gelatin (>98%, Sigma-Aldrich, Co., Ltd., St. Louis, MO, USA) in 30 mL of high-purity water was prepared by heating to 40 °C and then poured onto the diet and mixed for 5 min. The diets were then placed in an oven at 45 °C and dried overnight [55].

### 2.3. Fish Maintenance, Bioassay and Sampling

Juvenile Goldfish (*C. auratus*) specimens with an average length of 10.14 ± 2.36 cm and weight of 15.25 ± 0.51 g were purchased from a local breeding farm (Sangar County, Rasht, Iran). After a day’s acclimatization period, the fish were randomly assigned to 4 experimental groups, with 3 replicates per group in 12 aquariums (15 L; 10 fish per tank). The groups were as follows: Control group (0 mg/kg PS-NPs = T_0_), Group I (1 mg/kg PS-NPs = T_1_), Group II (10 mg/kg PS-NPs = T_10_), and Group III (100 mg/kg PS-NPs = T_100_). Each group as fed with a diet containing PS-NPs twice daily at 8:00 a.m. and 5:00 p.m. for 21 days with 5% of body weight. The aquariums were continuously aerated using an air compressor. According to the USEPA (2002) standard, this exposure period simulates chronic, long-term exposure to nano-pollutants [56]. Water physicochemical parameters were monitored daily to ensure water quality, including temperature (26 ± 1 °C), dissolved oxygen (4 mg/L), nitrates (<10 mg/L), nitrite (<0.5 mg/L), ammonia (<0.15 mg/L), and light conditions (12 Light:12 Dark). Every 48 h, the bottoms of the aquariums were cleaned, and one-third of the water was replaced with de-chlorinated water. After 21 days of exposure to the nanoparticle-containing diet, 9 fish from each group were euthanized by over-anesthetizing them in a bath of clove extract (50 mg/L), as described by Balamurugan et al. (2016) [57]. All experimental procedures were carried out in compliance with the 3Rs principles of animal testing (replacement, reduction, and refinement) and the Iranian regulations [58], which align with the International Guiding Principles for Biomedical Research Involving Animals (EU, 2010/63).

Fish blood sampling and necropsy were conducted following established protocols to collect tissues for oxidative stress biomarker and digestive enzyme measurements. Blood was collected from the caudal vein using sterile syringes and collected into heparinized tubes for plasma. For necropsy, the fish were placed on a sterile dissection board, and aseptic techniques were used to remove the liver, kidney, and gills for oxidative stress biomarker analysis, as well as the intestines for digestive enzyme measurements. The abdominal cavity was opened carefully, and tissues were excised, rinsed with ice-cold, Phosphate-Buffered Saline (PBS; 1X), pH 7.4 (Gibco Co., Carlsbad, CA, USA), and snap frozen in liquid nitrogen before being stored at −80 °C. Intestines were rinsed with PBS to clean the lumen before freezing.

### 2.4. The Oxidative Stress Biomarkers

Liver, kidney, and gill tissues were weighed and homogenized on ice using a homogenizer at a tissue-to-buffer ratio of 1:10 (e.g., 100 mg tissue in 1-mL ice-cold PBS or a specific homogenization buffer containing 1–2 mM EDTA disodium salt, ACS reagent, ≥99.0%—(Sigma-Aldrich Co., Ltd., St. Louis, MO, USA) and 0.1% Triton™ X-100, laboratory grade (Sigma-Aldrich Co., Ltd., St. Louis, MO, USA) X-100, optionally supplemented with a protease inhibitor cocktail). The homogenates were centrifuged at 15,000× *g* for 15 min at 4 °C to remove debris, and the supernatants were carefully transferred to pre-labeled sterile microcentrifuge tubes. These samples were immediately snap frozen in liquid nitrogen and stored at −80 °C for subsequent biochemical analyses. Catalase (CAT), superoxide dismutase (SOD), glutathione peroxidase (GPx), and malondialdehyde (MDA) activities were determined using diagnostic reagent kits (ZellBio, GmbH., Lonsee, Baden-Württemberg, Germany) according to the product instructions. Total protein content was determined using the colorimetric method (Biorad Protein Assay), by a diagnostic kit (Bionic Co., Tehran, Iran). All experimental methods and formulas strictly followed the manufacturers’ instructions.

### 2.5. Integrated Biomarker Response (IBR)

The Integrated Biomarker Response (IBR) was measured based on the mathematical methods presented by Beliaeff and Burgeot (2002) and Resende and Pereira (2024) [59,60]. In this study, the IBR results were used to represent the stress level of Goldfish exposed to four concentrations of NPs for 21 days by integrating the measured biomarker results (SOD, CAT, MDA, and GPX).

### 2.6. The Blood Biochemical Parameters

Blood samples were randomly collected from 9 fish in each tank. Next, samples were centrifuged at 4000× *g* for 10 min at 4 °C to separate plasma or serum. These were then transferred into labeled microcentrifuge tubes and stored at −80 °C for biochemical analyses.

The activities of lactate dehydrogenase (LDH), alanine aminotransferase (ALT), aspartate aminotransferase (AST), and alkaline phosphatase (ALP) enzymes were determined using commercially available quantitative detection kits (Pars Azmun Co., Alborz, Iran). All assays followed the manufacturer’s protocols to ensure accuracy and reliability. Absorbance values were measured at the designated wavelengths using a spectrophotometer to quantify enzyme activities.

### 2.7. The Digestive Enzyme Activities in Intestinal Tissue

Intestinal tissue was homogenized using the above-mentioned method. Homogenates were centrifuged at 15,000× *g* for 15 min at 4 °C, and supernatants were transferred to labeled tubes, snap frozen in liquid nitrogen, and stored at −80 °C for analysis. The activities of amylase, lipase, and protease in intestinal tissue were assessed using quantitative detection kits (Sigma-Aldrich Co., Ltd., St. Louis, MO, USA). All procedures were carried out strictly following the manufacturer’s instructions.

### 2.8. Data Analysis

Data were analyzed using IBM SPSS Statistics for Windows (Version 27.0.1, Armonk, NY, USA). The homogeneity of variances and the normality of data were tested with Levene’s test and the Shapiro–Wilk test, respectively. Data with normal distribution and equal variances were analyzed with a one-way ANOVA test and a post hoc Tukey’s test to determine statistical significance between groups. In the case of unequal variances between groups, the ANOVA-Welch test was used. Results are presented as bar graphs (created with GraphPad Prism 10 software) showing mean ± SD (n = 9) with a significance level of *p* < 0.05. Significant differences between groups are indicated by the following: * *p* < 0.05, ** *p* < 0.01, *** *p* < 0.001, **** *p* < 0.0001, and ns indicating non-significance. Calculations related to the IBR index were performed using the R software package IBRtools (Version 0.1.3) prepared by Resende and Pereira [60].

## 3. Results

### 3.1. Changes in Antioxidant Biomarkers in Gill Tissue

**Figure 1:**
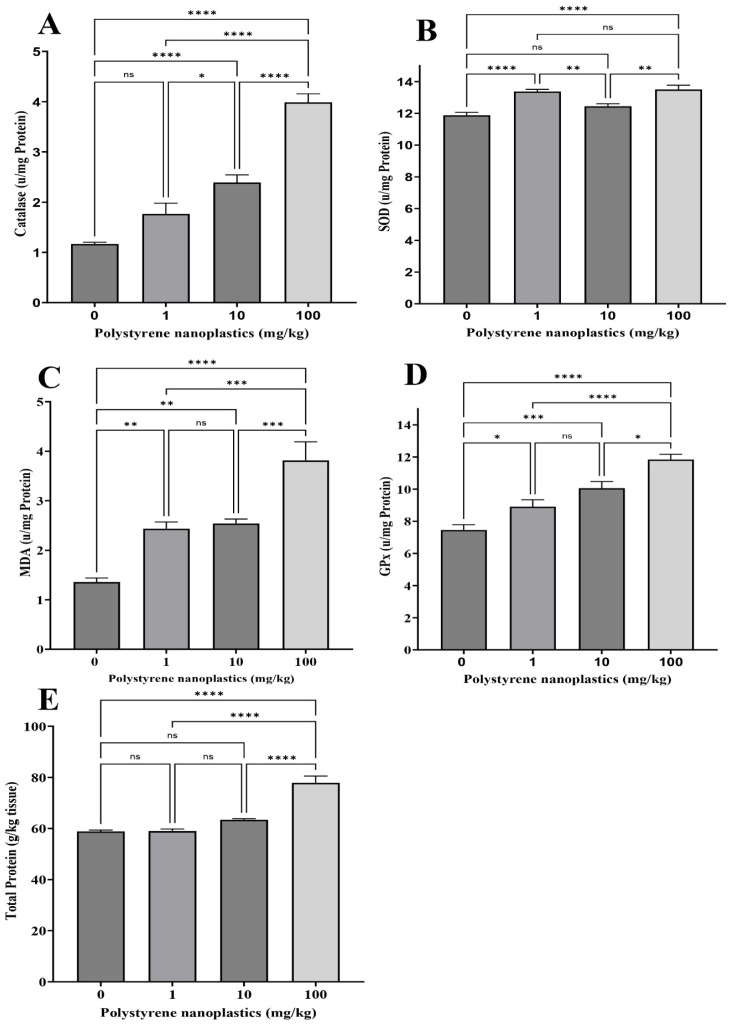
Oxidative stress biomarker activity: (**A**) CAT, (**B**) SOD, (**C**) MDA, (**D**) GPX, and (**E**) total protein in gills of Goldfish exposed to NPs. All data are presented as mean ± SD, * *p* < 0.05, ** *p* < 0.01, *** *p* < 0.001, **** *p* < 0.0001, and ns *p* > 0.05.

According to the results, catalase (CAT) activities were significantly higher in the gills of T_10_ and T_100_ compared to T_0_ (df = 3, *p* < 0.0001; F = 59.81 for both; Figure 1A). In contrast, no significant difference was observed in CAT activity in the gills of T_1_ and the control (df = 3, *p* = 0.051). Glutathione peroxidase (GPx) activity in the gills of T_100_, T_10_, and T_1_ were significantly higher than in T_0_ (df = 3, *p* < 0.0001; df = 3, *p* < 0.001, and df = 3, *p* = 0.049; F = 24.03, respectively; Figure 1B). Malondialdehyde (MDA) activity was significantly increased in T_100_, T_10_, and T_1_ compared to T_0_ (df = 3, *p* < 0.0001; df = 3, *p* = 0.002, and df = 3, *p* = 0.005; F = 23.10, respectively; Figure 1C). Although superoxide dismutase (SOD) activity was significantly higher in T_100_ and T_1_ than in the control group (df = 3, *p* < 0.0001; F = 15.98 for both), however, no significant alterations were found in its activity between T_10_ and the control (df = 3, *p* = 0.182; Figure 1D). The findings showed that oral exposure to PS-NPs significantly increased the total protein in T_100_ compared to T_0_ (df = 3, *p* < 0.0001; F = 39.41; Figure 1E). T_1_ and T_10_ showed no significant changes (df = 3, *p* = 1.000 and df = 3, *p* = 0.130, respectively.)

### 3.2. Changes in Antioxidant Biomarkers in Kidney Tissue

**Figure 2:**
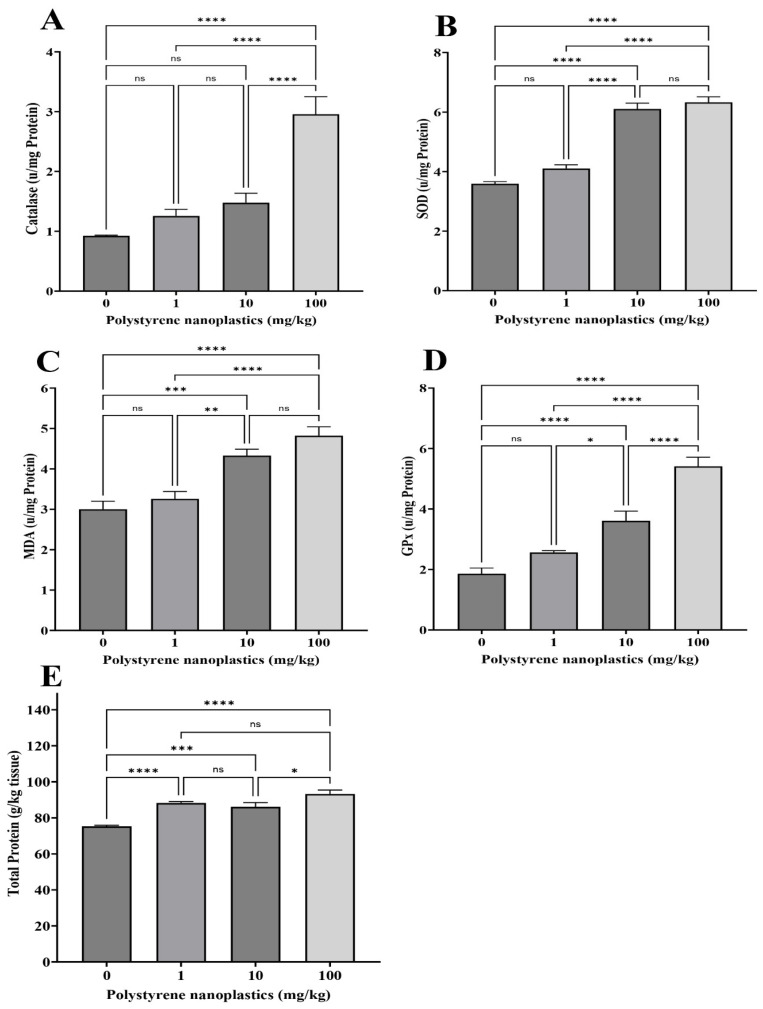
Oxidative stress biomarker activity: (**A**) CAT, (**B**) SOD, (**C**) MDA, (**D**) GPX, and (**E**) total protein in kidneys of Goldfish exposed to NPs. All data are presented as mean ± SD, * *p* < 0.05, ** *p* < 0.01, *** *p* < 0.001, **** *p* < 0.0001, and ns *p* > 0.05.

A significant change was observed in the CAT activity in the kidney tissue of T_100_ compared to of T_0_ (df = 3, *p* < 0.0001; F = 26.19; Figure 2A). On the other hand, no significant differences were found between some treatments (T_1_ and T_10_) and T_0_ (df = 3, *p* = 0.545 and df =3, *p* = 0.137, respectively). According to the results, significant elevation in GPx activity of the kidney was observed in T_10_ and T_100_ compared to T_0_ (df = 3, *p* < 0.0001; F = 40.83 for both; Figure 2B). No significant changes were observed between T_1_ and T_0_ (df = 3, *p* = 0.186). MDA activity is shown in Figure 2C, exhibiting that its activity in fish kidney cells was significantly upraised in T_10_ and T_100_ compared to T_0_ (df = 3, *p* < 0.0001 and df = 3, *p* < 0.001; F = 20.33, respectively). In contrast, no significant differences were observed between T_1_ and T_0_ (df = 3, *p* = 0.780). The highest MDA levels was observed in T_100_. Moreover, the findings revealed that MDA levels were dose-dependent. SOD activity in T_10_ and T_100_ was significantly higher than in T_0_ (df = 3, *p* < 0.0001; F = 84.20 for both; Figure 2D). No significant difference was observed between T_1_ and T_0_ (df = 3, *p* = 0.101). The results showed that exposure to different PS-NP doses significantly increased total protein in all treatments (T_1_, T_100_ and T_10_ compared to T_0_, respectively; df = 3, *p* < 0.0001; df = 3, *p* < 0.0001 and df = 3, *p* < 0.001; F = 20.56; Figure 2E). Moreover, alteration in total protein was also dose-dependent.

### 3.3. Changes in Antioxidant Biomarkers in the Liver Tissue

**Figure 3:**
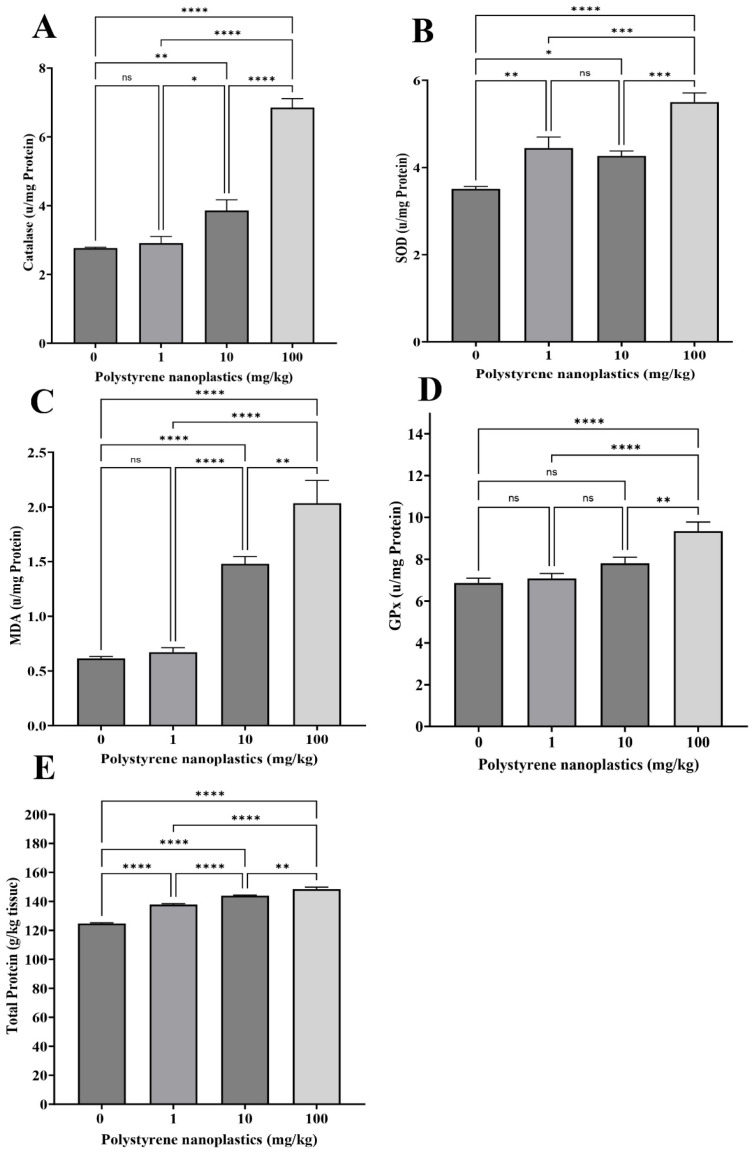
Oxidative stress biomarker activity: (**A**) CAT, (**B**) SOD, (**C**) MDA, (**D**) GPX, and (**E**) total protein in liver of Goldfish exposed to NPs. All data are presented as mean ± SD, * *p* < 0.05, ** *p* < 0.01, *** *p* < 0.001, **** *p* < 0.0001, and ns *p* > 0.05.

The CAT activity in the fish hepatocytes of T_100_ and T_10_ was significantly higher than in T_0_ (df = 3, *p* < 0.0001 and df = 3, *p* = 0.008; F = 72.37, respectively; Figure 3A). No significant differences were observed between T_1_ and T_0_ (df = 3, *p* = 0.968). According to the results, GPx activity was significantly higher in the fish liver of T_100_ than that of T_0_ (df = 3, *p* < 0.0001; F = 13.37; Figure 3D). No significant changes were observed between T_1_ and T_10_ (df = 3, *p* = 0.956; df = 3 and *p* = 0.151, respectively). MDA levels were significantly higher in the fish hepatocytes of T_100_ compared to T_0_ (df = 3, *p* < 0.0001; F = 37.24; Figure 3C). However, T_1_ showed no significant differences (df = 3, *p* = 0.983 and df = 3, *p* = 0.151, respectively) compared to the control group. The results showed that the activity of SOD in all treatments (T_100_, T_1_, and T_10_, respectively) was significantly higher than in the control group (df = 3, *p* < 0.0001; df = 3, *p* = 0.003 and df = 3, *p* = 0.022; F = 22.03; Figure 3B). The highest SOD activity was observed in the fish hepatocytes of T_100_ (Figure 3B). Significant alterations were also observed in the total protein of the hepatocytes in different treatments (df = 3, *p* < 0.0001; F = 165.69 for all; Figure 3E).

### 3.4. Changes in the Plasma Biochemical Parameters

**Figure 4:**
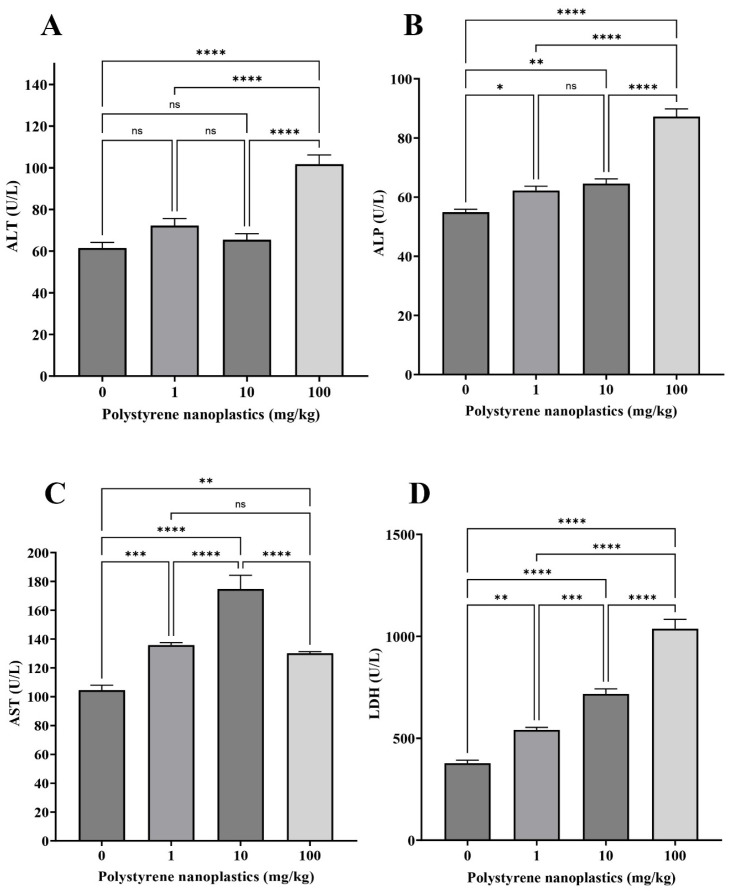
Biochemical enzyme activity: (**A**) ALP, (**B**) ALT, (**C**) AST, and (**D**) LDH in plasma of Goldfish exposed to NPs. All data are presented as mean ± SD, * *p* < 0.05, ** *p* < 0.01, *** *p* < 0.001, **** *p* < 0.0001, and ns *p* > 0.05.

The findings showed that oral administration of different doses (T_100_, T_10_ and T_1_) of PS-NPs significantly increased alkaline phosphatase (ALP) activity in plasma (df = 3, *p* < 0.0001; df = 3, *p* = 0.003 and df = 3, *p* = 0.031; F = 62.09, respectively; Figure 4A). Exposure to PS-NPs significantly increased alanine aminotransferase (ALT) activity in the fish plasma of T_100_ compared to T_0_ (df = 3, *p* < 0.0001; F = 28.31). In contrast, no significant differences were observed between some treatments (T_1_, T_10_) and T_0_ (df = 3, *p* = 0.139 and df = 3, *p* = 0.848 respectively; Figure 4B). A significant decrease was observed in the aspartate aminotransferase (AST) activity of different PS-NP treatments (T_10_, T_1_, and T_100,_ respectively, df = 3, *p* < 0.0001; df = 3, *p* < 0.001, and df = 3, *p* = 0.007; F = 31.50; Figure 4C). In contrast, lactate dehydrogenase (LDH) activity significantly elevated following fish exposure to various doses (T_100_, T_10_, and T_1_) of PS-NPs compared to T_0_ (df = 3, *p* < 0.0001; df = 3, *p* < 0.0001, and df = 3, *p* = 0.001; F = 102.77, respectively; Figure 4D).

### 3.5. IBR Index for Antioxidant Biomarkers in Liver, Kidney, and Gill Tissues

**Figure 5:**
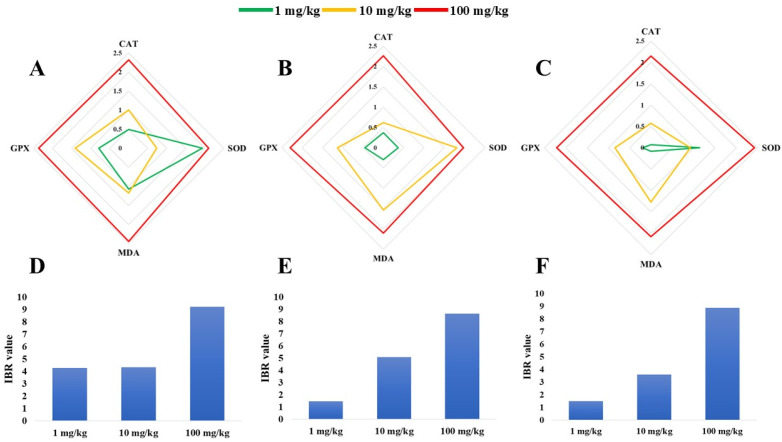
Radar plots of IBR in gill (**A**), kidney (**B**), and liver (**C**). IBR index of all tested biomarkers in gill (**D**), kidney (**E**), and liver (**F**).

According to radar plots, the highest IBR index of gill tissues was related to the MDA biomarker in T_100_ (Figure 5A). Similarly, the IBR index of the GPx biomarker was increased in T_100_. In addition, in the case of the SOD biomarker in the gills of the fish, the highest IBR was observed in T_1_. In kidney tissue (Figure 5B), the highest elevation in the IBR indexes were observed in the GPx, SOD, and GPx biomarkers assayed in T_100_, T_10_, and T_1,_ respectively. Similarly, in liver tissue (Figure 5C), increased IBR indexes were found in the SOD, MDA, and SOD biomarkers of T_100_, T_10_, and T_1_, respectively. The results showed that the sum of the IBR values of SOD, CAT, MDA, and GPx in the PS-NP-treated groups were higher than in the control group. The highest mean value (IBR = 9.23) in the gill tissue was observed in T_100_ (Figure 5D). In contrast, the lowest mean value (IBR = 1.49) in the fish kidney was recorded in T_1_ (Figure 5E).

### 3.6. Changes in Digestive Enzyme Activities in the Intestinal Tissue

**Figure 6:**
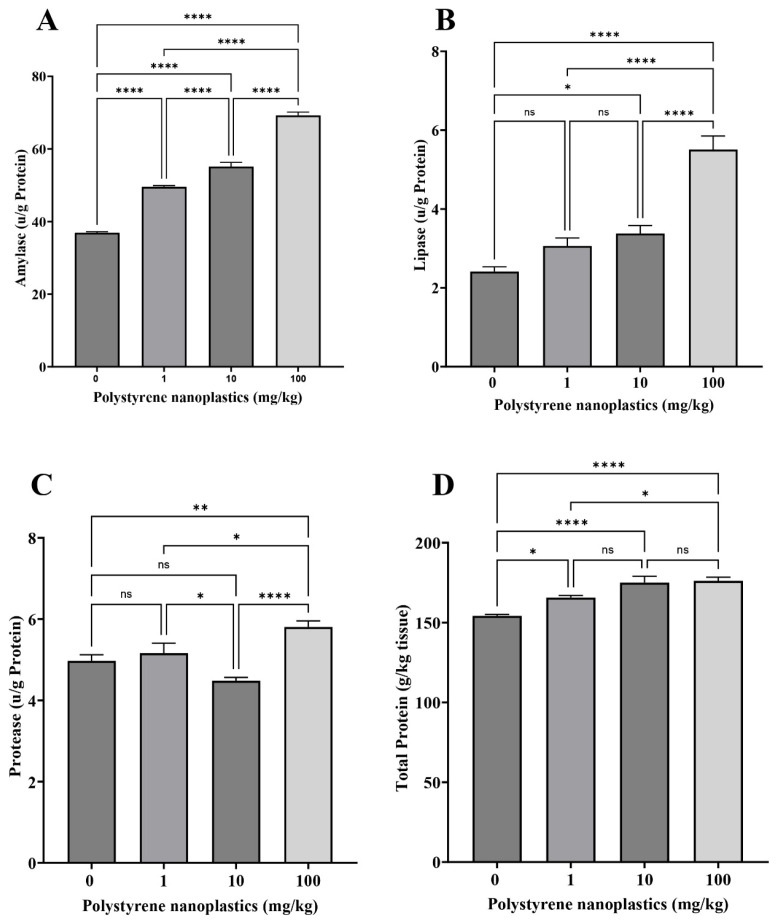
Digestive enzyme activity: (**A**) amylase, (**B**) lipase, (**C**) protease and (**D**) total protein in intestine tissue of Goldfish exposed to NPs. All data are presented as mean ± SD, * *p* < 0.05, ** *p* < 0.01, **** *p* < 0.0001, and ns *p* > 0.05.

The findings showed that oral administration of different doses of PS-NPs significantly elevated amylase activity in the fish intestine compared to the control group (df = 3, *p* < 0.0001; F = 316.81 for all; Figure 6A). Significant increases in lipase activity were observed in T_100_ and T_10_ compared to the control group (df = 3, *p* < 0.0001 and df = 3, *p* = 0.030; F = 33.16, respectively). No significant difference was detected between T_1_ and the control group (df = 3, *p* = 0.220; Figure 6B). In addition, protease activity was significantly influenced by T_100_ (df = 3, *p* = 0.007; F = 10.37). On the other hand, no significant difference was observed between T_1_ and T_10_ (df = 3, *p* = 0.858 and df = 3, *p* = 0.180, respectively; Figure 6C). The results showed that total protein activity in all treatments (T_100_, T_10_, and T_1_) were significantly higher than in the control group (T_0_), respectively (df = 3, *p* < 0.0001; df = 3, *p* < 0.0001 and df = 3, *p* = 0.013; F = 16.94; Figure 6D).

## 4. Discussion

Studies have shown that plastic debris, including micro-and nanoplastics (MNPs) present in both freshwater and marine ecosystems, could significantly affect fish biomass [61,62], fish physiology, and their survival [44,63,64,65]. These particles, especially NPs, can be uptaken by aquatic organisms, accumulate in digestive systems, and penetrate vital organs through biological liquids such as blood [35]. These processes could increase the bioaccumulation rate of NPs in various organs, such as the liver, gills, kidneys, and brain [35]. Therefore, cells try to follow different pathways to transform and detoxify these xenobiotics [3]. Studies have shown that cellular antioxidant defense mechanisms play a critical role in mitigating NP toxicity [35,66].

A significant increase in SOD activity in different tissues could facilitate biodegrading superoxide anions to H_2_O_2_ [67,68]. A significant increase in superoxide dismutase (SOD) activity in various tissues suggests an enhanced capacity to catalyze the dismutation of superoxide anions into hydrogen peroxide (H_2_O_2_) [67,68]. In the present study, exposure to polystyrene nanoplastics (PS-NPs) was found to induce SOD activity in fish cells. This upregulation likely represents a cellular antioxidant defense mechanism aimed at neutralizing superoxide anions generated by oxidative stress following PS-NP exposure. The increased production of H_2_O_2_ as a byproduct of this reaction is expected to subsequently trigger elevated activities of catalase (CAT) and glutathione peroxidase (GPx), which are responsible for H_2_O_2_ detoxification. A significant increase in CAT activity in the different fish tissues indicated a physiological cellular response to increased H_2_O_2_ following exposure to PS-NPs. Increasing CAT activity could degrade H_2_O_2_ in the cells to H_2_O and O_2_ [67,69]. Similarly, GPx catalyzes reduction of H_2_O_2_ and other reactive oxygen species (ROS) by conjugating them with glutathione (GSH), further contributing to cellular detoxification [70]. The observed increase in GPx activity confirms its role in neutralizing H_2_O_2_ within various organs.

Increased CAT and SOD activities were reported in the gills of Goldfish (*Carassius auratus*) in response to the increased activity of superoxide anions and hydrogen peroxide [71]. Furthermore, Liu et al. (2018) found that alterations in GPx activity might indicate cellular reactions to the elevated ROS [72]. A significant rise in CAT and SOD activities has been reported in the hepatocytes of *Pseudobagrus fulvidraco* [73]. Chen et al. (2022) and Pei et al. (2022) reported that elevated SOD and CAT activities in the hepatocytes of *D. rerio* and *Micropterus salmoides* could induce oxidative stress and activate the antioxidant defense system [68,74]. Similarly, a significant rise in SOD and CAT activities was found in the hepatocytes of *Oryzias melastigma* exposed to 10 µg/mL PS-NPs [75]. In addition, a significant upraise in GPx, SOD, and CAT activities was observed in the hepatocytes of *Sparus aurata* [76] and *Ietalurus punetaus* [77] after exposure to MNPs. Moreover, similar studies have shown that exposure to different concentrations of PS-NPs could significantly alter CAT, SOD, and GPx activities and MDA activity in *Larimichthys crocea* [35], zebrafish, *D. rerio* [78,79,80,81], European sea bass, *Dicentrarchus labrax* [82], sheepshead minnow, *Cyprinodon variegatus* [83], and medaka (*Oryzias latipes*) [80]. The gills are directly exposed to environmental pollutants. Therefore, oxidative reactions in the gills of fish may be sharper than in the other tissues. Lee et al. (2023), Li et al. (2023), and Sun et al., (2024) reported changes in antioxidant enzyme activities in the gills of *Pseudobagrus fulvidraco*, *D. rerio*, and mussel *Chlamys farreri* exposed to different concentrations of MNPs [73,79,84].

In the present study, after fish exposure to PS-NPs, although the SOD, CAT, and GPx activities were elevated, however, the cellular antioxidant capacity significantly dropped. In addition, decreased total antioxidant capacity is evidence of the collapse in cellular antioxidant defense. Elevated MDA reflects oxidative stress in fish exposed to PS-NPs. MDA is a primary metabolite produced in the peroxidation of polyunsaturated fatty acids, such as arachidonic acid, and is used as a reliable biomarker for an oxidative stress assay [80,85,86]. A significant elevation in MDA showed that the activity of different ROS significantly increased after fish exposure to PS-NPs, and the antioxidant defense system could neutralize ROS. Alterations in MDA activity in different tissues have been reported in medaka (*O. latipes*) [87], *S. aurata* [76], and zebrafish (*D. rerio*) [33] following exposure to MNPs. Gómez-Zubeldia et al. (2000) and Xia et al. (2020) believed that MDA activity indirectly reflects oxidative damage [88,89].

The IBR index effectively integrates biomarker response values [59] It is used to assess an organism’s health status under environmental stress [90]. The IBR index showed that exposure to different concentrations of PS-NPs could induce oxidative damage to various fish tissues. A higher IBR value indicates more significant toxicity to the organism under those environmental conditions. In this study, the IBR values of fish exposed to PS-NPs were higher than those of the control group, indicating the integrated biological responses and poor health conditions in the exposed Goldfish. The highest IBR index was found in T_100_ and T_10_, respectively. Furthermore, these findings demonstrate that alterations in the IBR index due to PS-NPs are dose-dependent. Similar changes in the IBR index have been reported in *Larimichthys crocea* [35] and guppy fish (*Poecilia reticulata*) [91] exposed to NPs and MPs.

Elevated lipid peroxidation in the cells challenged by PS-NPs could change cellular stability and permeability. Therefore, upraised AST, ALT, ALP, and LDH activities in the plasma of the fish exposed to PS-NPs is evidence of damage to the cellular membrane. Moreover, these enzyme activities are biomarkers of cellular health. They play a critical role in different cellular biochemical reactions. Alterations in ALT and AST activities can affect amino transformation in cells. Thus, upraised AST and ALT activities in cells can help to supply energy by amino transformation [92]. Changes in ALP activity may alter phosphorylation biochemical reactions in cells [93]. Moreover, elevated ALP activity can manage inflammation conditions through the induced anti-inflammation mechanisms [94]. Studies have shown that exposure to different xenobiotics forces cells to shift the process of energy production from aerobic to anaerobic metabolism. In anaerobic conditions, lactate may be increased. Thus, increased LDH activity is necessary to biodegrade extra lactate. In addition, increased LDH activity may be due to necrosis or cell death [93]. The release of LDH into the bloodstream may be in association with the destruction of the cell membrane structure. Therefore, alterations in LDH activities may be related to cellular hypoxia and mitochondrial oxidative dysfunction [93,94]. Consequently, increased AST, ALT, ALP, and LDH activities showed cellular physiological dysfunctions. Significant elevations in AST, ALT, ALP, and LDH activities have been reported in *O. niloticus* [95], *D. rerio* [96], *Cyprinus carpio* [97,98], and African catfish (*Clarias gariepinus*) exposed to different concentrations of MPs [99]. They showed that alterations in these enzymes are evidence of oxidative damage to the cellular membrane. Similarly, scientists have shown that exposure to MNPs upraised AST, ALT, ALP, and LDH activities in *Larimichthys crocea* [100], *Pseudobagrus fulvidraco* [73], and *C. carpio* [92]. Moreover, a significant elevation in LDH activity was observed in discus fish following exposure to MPs [101]. Similar results were reported in fish exposed to plastic polymer softeners [102].

The adverse effects of PS-NPs observed in Goldfish (*Carassius auratus*) in this study, including oxidative stress, cellular damage, and altered digestive enzyme activities, suggest multiple toxicity mechanisms, particularly involving interactions with cells and biological membranes. Owing to their nanoscale size (50 nm in this study), PS-NPs exhibit a high surface area-to-volume ratio and enhanced reactivity, facilitating their penetration into biological systems [10]. These properties may enable PS-NPs to interact with cellular membranes through physical adsorption, hydrophobic interactions, or direct penetration, potentially disrupting membrane integrity and function [1,53]. Our findings of significantly elevated plasma enzymes, such as AST, ALT, ALP, and LDH, in fish exposed to PS-NPs support this hypothesis, indicating that these nanoparticles compromise cellular membrane stability, leading to enzyme leakage into the bloodstream. This disruption is likely linked to lipid peroxidation, as evidenced by increased MDA levels in gill, kidney, and liver tissues, which reflects oxidative damage to polyunsaturated fatty acids within membranes [85,88].

The induction of oxidative stress by PS-NPs, demonstrated by elevated activities of antioxidant enzymes (SOD, CAT, and GPx) and MDA (Section 3.1, Section 3.2 and Section 3.3), points to a mechanism involving reactive oxygen species (ROS) generation. The small size and surface properties of PS-NPs may allow them to interact with intracellular organelles, such as mitochondria or the endoplasmic reticulum, triggering ROS production [24,66]. This oxidative burden could overwhelm the cellular antioxidant defense system, as suggested by the increased enzyme activities failing to prevent elevated MDA levels, indicating a collapse in total antioxidant capacity [69]. The dose-dependent rise in MDA and IBR index values further suggests that higher PS-NP concentrations intensify these effects, likely due to increased particle uptake and cellular interactions [33,35]. Such oxidative stress aligns with reports on other fish species, where PS-NPs induce ROS-mediated damage in hepatocytes and gills [15,78].

Additionally, PS-NPs may exert toxicity through direct physical interactions with biological membranes, altering fluidity and permeability. Studies have shown that nanoplastics can embed within or translocate across lipid bilayers, potentially disrupting ion transport, nutrient uptake, and cellular signaling [14,30,64]. In our study, the altered digestive enzyme activities (amylase, lipase, and protease) in intestinal tissue could be associated with such membrane interactions, where PS-NPs impair the gastrointestinal epithelial barrier, hinder nutrient absorption, or interfere with enzyme secretion [103,104]. This is consistent with the accumulation of NPs in the digestive system and their subsequent penetration into vital organs via biological fluids, as noted in the Introduction section [16,20].

Moreover, PS-NPs may interact with membrane proteins and receptors, potentially disrupting cellular signaling and immune responses. The elevated total protein levels in exposed tissues could reflect compensatory protein synthesis or stress responses triggered by such interactions [23,25]. For instance, PS-NPs might adsorb onto membrane-bound enzymes or transporters, altering their function and contributing to the observed physiological dysfunctions. While our study did not directly assess these molecular interactions, the biochemical and histological changes align with mechanisms reported in the literature, such as immunomodulation and membrane destabilization in aquatic organisms exposed to nanoplastics [27,51]. These interactions could also facilitate the translocation of PS-NPs across barriers like the blood–brain barrier, as observed in other studies [39], posing additional risks to neurological health.

In conclusion, the toxicity of PS-NPs in Goldfish likely arises from their capacity to penetrate and destabilize biological membranes, generate ROS-mediated oxidative stress, and interfere with cellular processes through physical and biochemical interactions. These mechanisms collectively underpin the observed physiological and biochemical alterations, emphasizing the need for further research to elucidate the specific molecular pathways and long-term implications of PS-NP exposure in aquatic organisms.

MPs and NPs may damage the gastrointestinal tract of fish by causing physical abrasion, inducing inflammation, or disrupting epithelial integrity, potentially leading to malnutrition and developmental problems [103,104]. Studies have shown that oral exposure to MPs could affect digestive enzyme activities in the intestinal tissue. Therefore, monitoring the activities of amylase, lipase, and protease could help to assay the toxicity of PS-NPs in Goldfish. The results showed that alterations in these enzymes due to PS-NPs were dose-dependent. Elevated amylase, lipase, and protease activities may be a reaction to facilitate the elimination of NPs from the digestive system. Frank et al. (2023) reported that exposure to PS-MPs for 1–6 days could increase amylase activity in *Coregonus peled* larvae [105]. The elevation in specific pancreatic enzyme activity in this study may be related to impaired nutrient absorption due to the effect of MPs on the fish gastrointestinal tract. A significant rise in lipase and amylase activities have been observed in *Coreius guichenoti* exposed to 1000 μg/L polyethylene MPs [106]. Similarly, Barus et al. (2023) and Trestrail et al. (2021) reported a significant upraise in the protease and amylase activities of the mussels *Paphia undulata* and *Mytilus galloprovincialis*, respectively, following exposure to PS-MPs, [107,108]. Similar to our results, stimulation of digestive enzymes in the presence of 200–1000 μg/L polyvinyl chloride (PVC) was observed in juvenile freshwater fish *Barbodes gonionotus* (Silver barb) after 96 h [71]. In contrast, the activities of lipase, protease, and amylase significantly dropped in the digestive system of *Oryzias latipes* [109], juvenile guppies [104], and *Nothobranchius guentheri* [110] exposed to MNPs.

## 5. Conclusions

In this study, exposure to dietary NPs could induce oxidative stress, alteration in blood biochemical parameters, and digestive enzyme activities in Goldfish. This study showed that chronic exposure to NPs adversely affected the health of the gills, kidneys, liver, and intestinal tissues. Alterations in oxidative biomarkers in fish exposed to different concentrations of PS-NPs exhibited the potential for toxicity of this polymer to aquatic organisms. Moreover, changes in the plasma enzyme activities displayed that PS-NPs could damage cellular membranes and disturb cell biochemical reactions. In addition, alterations in the digestive enzymes suggested that oral exposure to PS-NPs could affect the digestive system functions of the fish. Our findings highlight the toxicological risks of dietary PS-NP exposure, particularly its effects on oxidative stress, blood biochemistry, and digestion in freshwater fish. Our results highlight that exposure to PS-NPs could affect fish health, suggesting that further research is needed to assay different aspects of NP toxicity in fish, such as the following: (1) investigating the long-term bioaccumulation and trophic transfer of PS-NPs in aquatic food webs to assess ecosystem-level impacts, given their penetration into vital organs; (2) examining the molecular mechanisms (e.g., gene expression of antioxidant enzymes or inflammatory pathways) underlying the observed oxidative stress and digestive enzyme alterations to clarify PS-NP toxicity pathways; and (3) evaluating the combined effects of PS-NPs with other environmental pollutants (e.g., heavy metals or organic contaminants) to reflect realistic exposure scenarios, as fish in natural settings are rarely exposed to NPs in isolation Therefore, further studies are necessary to understand the extent of these nano-pollutants impacts on freshwater ecosystems. These findings contribute to improving knowledge about the adverse effects of PS-NPs on fish health and nutritional quality by demonstrating the following: (1) the dose-dependent induction of oxidative stress in multiple organs (liver, kidney, gills), as evidenced by elevated antioxidant enzyme activities and lipid peroxidation; (2) the systemic impact on blood biochemical parameters, indicating cellular damage and physiological dysfunction; and (3) the alteration of digestive enzyme activities, suggesting potential nutritional impairments in Goldfish.

## Data Availability

The original contributions presented in this study are included in the article/Supplementary Material. Further inquiries can be directed to the corresponding authors.

## Supplementary Materials

The following supporting information can be downloaded at: https://www.mdpi.com/article/10.3390/toxics13050336/s1, Figure S1: SEM Image Interpretation of Polystyrene Nanoplastics; Figure S2: Micro Fourier Transform Infrared (Micro-FTIR) spectroscopy spectrum for polystyrene nanoplastics Table S1: Chemical and physical characteristics of Polystyrene nanoplastics suspension.

## Abbreviations

The following abbreviations are used in this manuscript:

NP	Nanoplastic
MP	Microplastic
PS	Polystyrene
*C. auratus*	*Carassius auratus*
T_0_	Treatment 0 mg/kg
T_1_	Treatment 1 mg/kg
T_10_	Treatment 10 mg/kg
T_100_	Treatment 100 mg/kg
AST	aspartate aminotransferase
ALT	alanine aminotransferase
ALP	alkaline phosphatase
LDH	lactate dehydrogenase
GPx	glutathione peroxidase
CAT	catalase
SOD	superoxide dismutase
GSH	glutathione
MDA	malondialdehyde
ROS	reactive oxygen species
IBR	Integrated Biomarker Response
EDTA	Ethylene Diamine Tetraacetic Acid
μ-FT-IR	Micro Fourier Transform InfraRed spectroscopy
DLS	dynamic light scattering
PBS	Phosphate-Buffered Saline
