# Supplementary material for: Effects of Polystyrene Nanoplastics on Oxidative Stress, Blood Biochemistry, and Digestive Enzyme Activity in Goldfish (Carassius auratus)
Source: Toxics. 2025 Apr 24;13(5):336. doi: 10.3390/toxics13050336 (PMC12115821; doi:10.3390/toxics13050336)
Supplement: Supplementary file 1 [file toxics-13-00336-s001.zip › toxics-3571296-supplementary.pdf]

Table S1. Chemical and physical characteristics of Polystyrene nanoplastics suspension.

Parameters	Values
Dynamic Light Scattering (DLS)	~50 nm
Zeta potential	-160 mV
Scanning Electron Microscope (SEM)	Figure A1
Micro Fourier Transform Interferometer (μ-FT-IR)	Figure A2

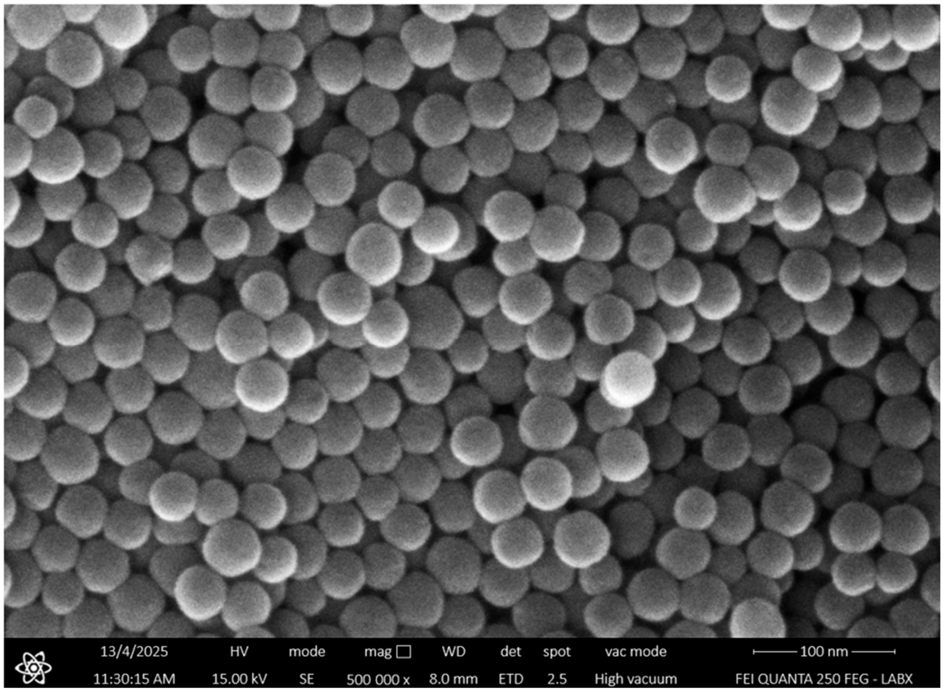

Figure S1: SEM Image Interpretation of Polystyrene Nanoplastics

Observed Features:

1. The particles are **spherical** and show a **smooth surface** texture, which is typical of well-synthesized polystyrene nanoplastics.
2. Uniform shape indicates **monodispersity**, which is ideal for toxicological studies as it reduces variability.
3. Based on the scale bar (100 nm), the **diameter** of the particles appears to be in the range of approximately **50 nm**.
4. The particles are densely packed and closely associated, possibly due to **drying-induced aggregation** or **electrostatic interactions** during sample prep.

5. However, individual particles are still distinguishable, which helps in analyzing size and surface characteristics.

No visible porosity or irregular textures—surfaces appear **highly smooth**, suggesting no surface modifications or coatings (unless invisible at this resolution).

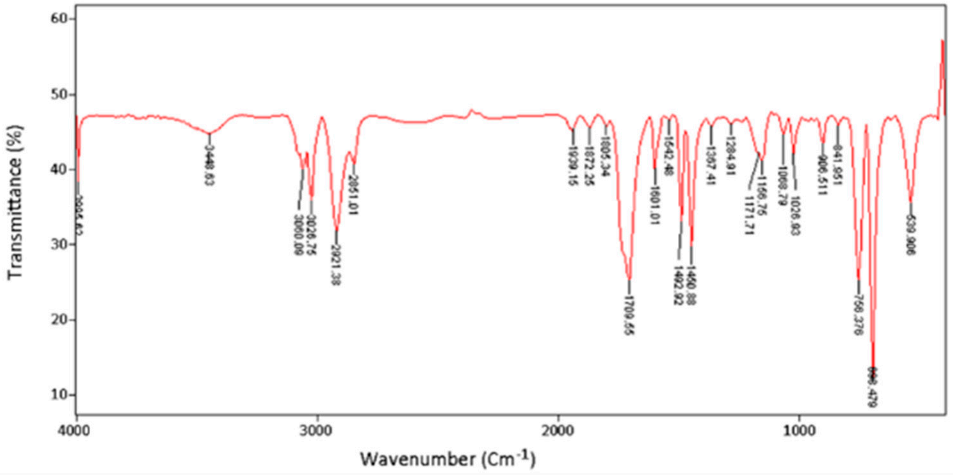

Figure S2: Micro Fourier Transform Infrared (Micro-FTIR) spectroscopy spectrum for polystyrene nanoplastics

Summary of Polystyrene Nanoplastics in This Spectrum

The FTIR spectrum confirms the presence of polystyrene through its characteristic peaks:

- Aromatic C-H stretching (3025–3060 cm^{-1})
- Aliphatic C-H stretching (2850–2925 cm^{-1})
- Aromatic C=C stretching (1601, 1493 cm^{-1})
- C-H bending (1452 cm^{-1} and 698–756 cm^{-1})

These peaks align with the molecular structure of polystyrene, which consists of a benzene ring (aromatic) and a hydrocarbon backbone (aliphatic). The spectrum is a good match for polystyrene nanoplastics, with no obvious signs of significant contamination or degradation.
